# Correction: Variation in the incidence of type 1 diabetes mellitus in children and adolescents by world region and country income group: A scoping review

**DOI:** 10.1371/journal.pgph.0003398

**Published:** 2024-06-14

**Authors:** The PLOS Global Public Health Staff

There are errors in the author affiliations. The correct affiliations are as follows:

Apoorva Gomber^1^, Zachary J. Ward^2^, Carlo Ross^1,3^, Maira Owais^1,4^, Carol Mita^5^, Jennifer M. Yeh^6^, Ché L. Reddy^1^, Rifat Atun^1,7^

**1** Department of Global Health and Population, Harvard T.H. Chan School of Public Health, Boston, Massachusetts, United States of America **2** Center for Health Decision, Harvard T.H. Chan School of Public Health, Boston, Massachusetts, United States of America **3** Manchester University NHS Foundation Trust, Manchester, United Kingdom **4** Department of Biology, Department of Economics, Amherst College, Amherst, Massachusetts, United States of America **5** Countway Library, Harvard Medical School, Boston, Massachusetts, United States of America **6** Division of General Pediatrics, Boston Children’s Hospital, Harvard Medical School, Boston, Massachusetts, United States of America **7** Department of Global Health and Social Medicine, Harvard Medical School, Boston, Massachusetts, United States of America.

The publisher apologizes for the error.
