# Supplementary material for: Variation in the incidence of type 1 diabetes mellitus in children and adolescents by world region and country income group: A scoping review
Source: PLOS Glob Public Health. 2022 Nov 9;2(11):e0001099. doi: 10.1371/journal.pgph.0001099 (PMC10021400; doi:10.1371/journal.pgph.0001099)
Supplement: S1 Text — (PDF) [file pgph.0001099.s001.pdf]

Supplementary Material
(labelled as S1 Text)

Contents **Page**

Figures

Fig A: 2
Distribution of published T1D incidence studies by country from 1990-2021

Fig B: 3
Published T1D incidence studies by global, regional and national scale and type of data

Fig C: 4
Incidence rates of T1D by sex (0-14 years) per region

Fig D: 5
Secular trends of T1D incidence over time for age groups 0-14 years

Tables

Table A: Search strategy: concepts and controlled vocabulary 6

Table B: Search results summary 7

Table C: Data extraction template 8

PRISMA-ScR Checklist 9

Fig A: Distribution of published T1D incidence studies by country from 1990-2021

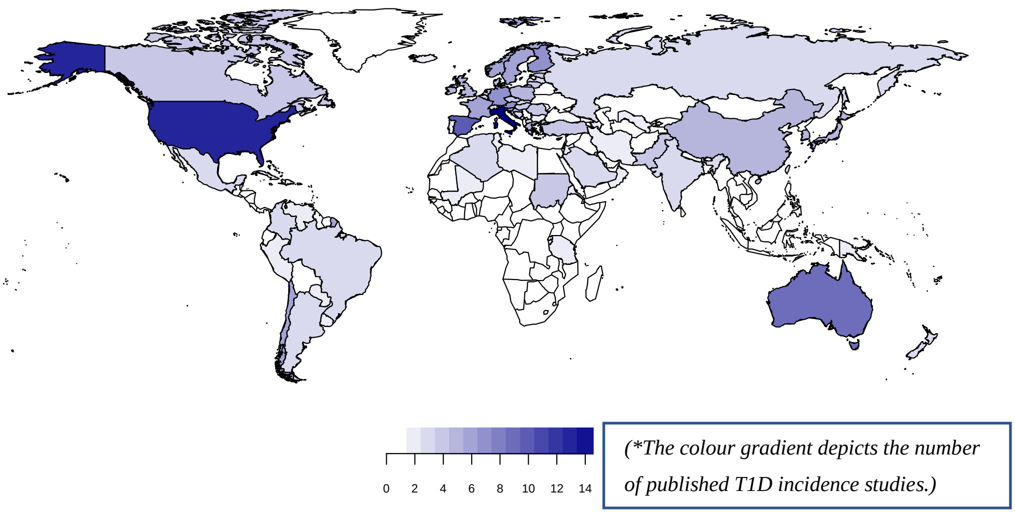

Source: Authors (created using maps package in R: https://cran.r-project.org/web/packages/maps/index.html)

Fig B: Published T1D incidence studies by global, regional and national scale and type of data

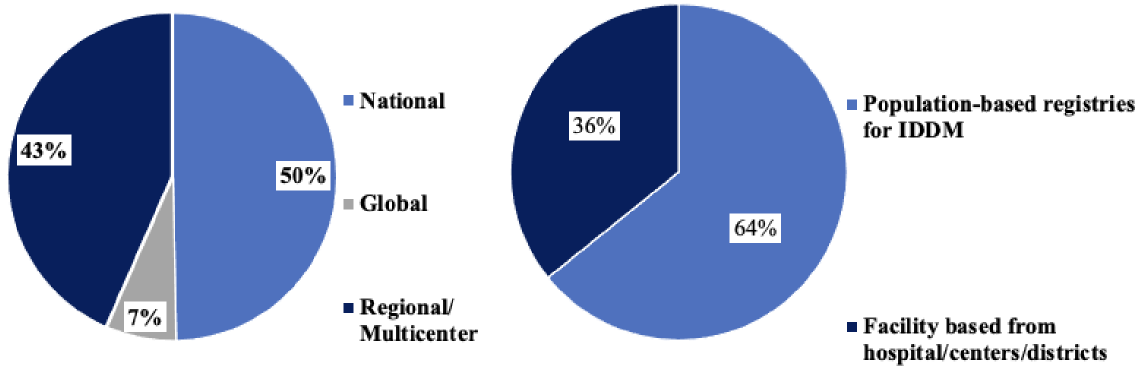

Fig C: Incidence rates of T1D by sex (0-14 years) per region

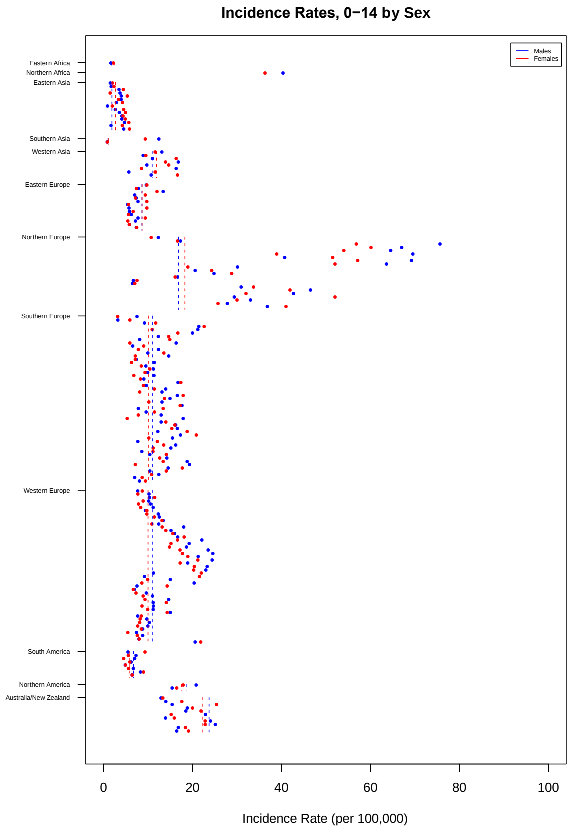

[Point sizes are proportional to their weight (i.e. inverse variance). The lines indicate means and shaded areas indicate 95% CIs.]

Fig D: Secular trends of T1D incidence over time for age groups 0-14 years

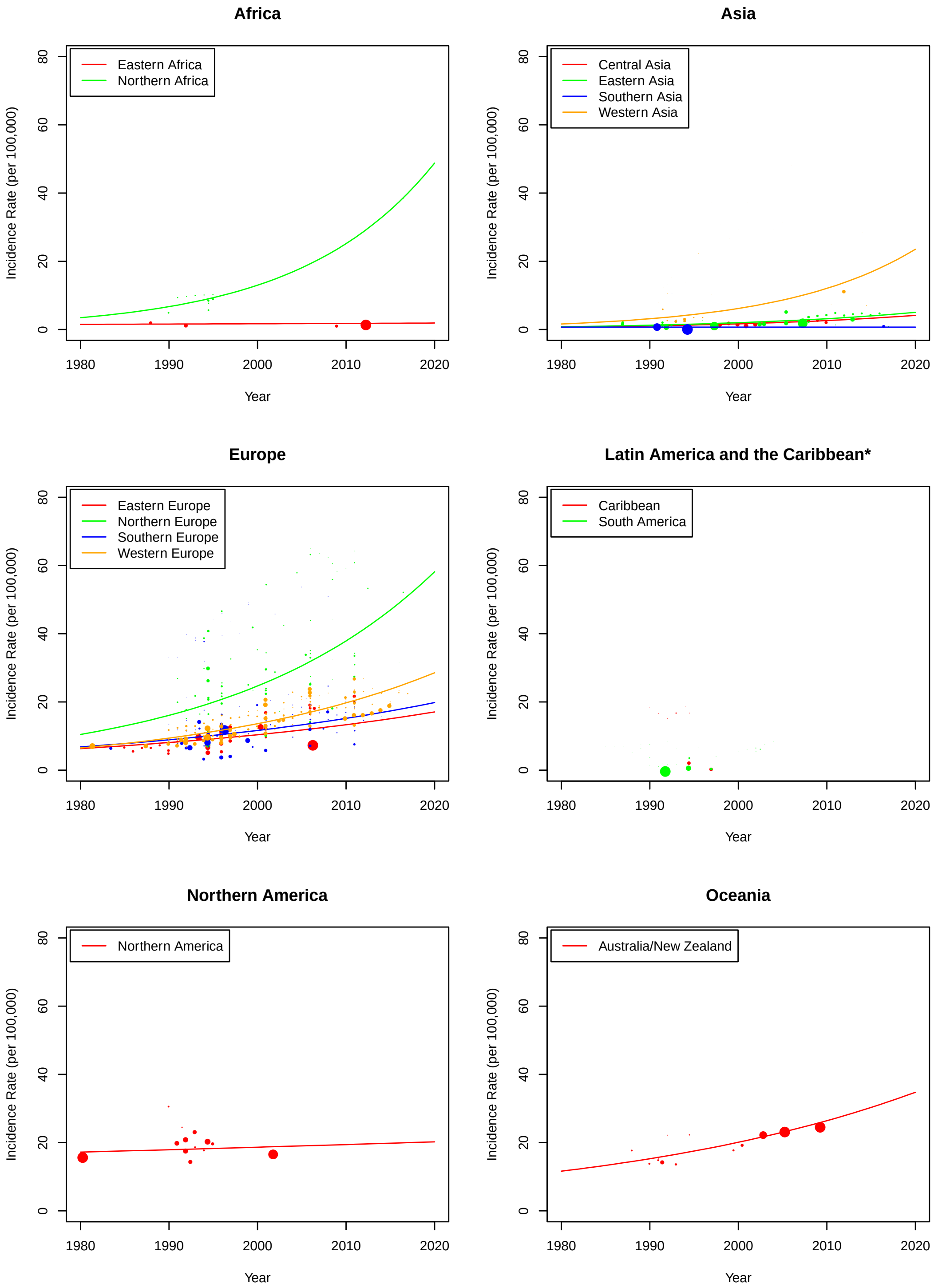

[Point sizes are proportional to their weight (i.e. inverse variance). The lines indicate means and shaded areas indicate 95% CIs.]

Table A: Search strategy: concepts and controlled vocabulary

<table>
<tr><th></th><th>Problem:
Type 1 Diabetes Mellitus</th><th>Incidence</th><th>Population:</th><th>Publication date:</th></tr>
<tr><td>Controlled vocabulary Terms [MeSH]</td><td>"Diabetes Mellitus, Type 1"</td><td>"Incidence"</td><td>"Infant"
"Child"
"Adolescent"
"Young Adult"</td><td>1990[pdat]:
2021[pdat]</td></tr>
<tr><td>Synonyms (tiab)</td><td>diabetes
AND
type 1
type I
childhood juvenile-onset
autoimmune
autoimmune
insulin-dependent</td><td>incidence
OR
registry</td><td>neonat*
new born*
newborn*
infancy
Infant
Infants
pre school
pre schooler*
preschool
preschooler*
child*
school age* school age*
preadolescen*
puberty
adolescen*
early adulthood
young adult*</td><td></td></tr>
<tr><td colspan="5">Combination Search:
("Diabetes Mellitus, Type 1"[Mesh] OR (diabetes[tiab] AND (type 1[tiab] OR type I[tiab] OR childhood[tiab] OR juvenile onset[tiab] OR autoimmune[tiab] OR auto immune[tiab] OR insulin-dependent[tiab])))

AND

("Incidence"[Mesh] OR incidence[tiab] OR registry[tiab])

AND

("Infant"[Mesh] OR "Child"[Mesh] OR "Adolescent"[Mesh] OR "Young Adult"[Mesh] OR neonat*[tiab] OR new born*[tiab] OR newborn*[tiab] OR infancy[tiab] OR infant[tiab] OR infants[tiab] OR pre school[tiab] OR pre schooler*[tiab] OR preschool[tiab] OR preschooler*[tiab] OR child*[tiab] OR school age*[tiab] OR schoolage*[tiab] OR preadolescen*[tiab] OR puberty[tiab] OR adolescen*[tiab] OR early adulthood[tiab] OR young adult*[tiab])

AND

(1990[pdat] : 2021[pdat])</td></tr>
</table>

Table B: Search results summary

(a) PubMed / Medline (National Library of Medicine, NCBI)= 4,527 results on 04/12/2021<br>("Diabetes Mellitus, Type 1"[Mesh] OR (diabetes[tiab] AND (type 1[tiab] OR type I[tiab] OR childhood[tiab] OR juvenile onset[tiab] OR autoimmune[tiab] OR auto immune[tiab] OR insulin-dependent[tiab])))<br>AND<br>("Incidence"[Mesh] OR incidence[tiab] OR registry[tiab])<br>AND<br>("Infant"[Mesh] OR "Child"[Mesh] OR "Adolescent"[Mesh] OR "Young Adult"[Mesh] OR neonat*[tiab] OR new born*[tiab] OR newborn*[tiab] OR infancy[tiab] OR infant[tiab] OR infants[tiab] OR pre school[tiab] OR pre schooler*[tiab] OR preschool[tiab] OR preschooler*[tiab] OR child*[tiab] OR school age*[tiab] OR schoolage*[tiab] OR preadolescen*[tiab] OR puberty[tiab] OR adolescen*[tiab] OR early adulthood[tiab] OR young adult*[tiab])<br>AND<br>(1990[pdat] : 2021[pdat])
(b) Embase (Elsevier, Embase-com)= 2,484 results 04/12/2021<br>Settings: Advanced Search<br>removing mapping options<br>Source: Embase<br>Date Limits: Publication years 1990-2021<br>publication types: article<br>1) 'insulin dependent diabetes mellitus'/exp<br>2) (diabetes NEAR/1 ('type 1' OR 'type I' OR childhood OR 'juvenile onset' OR autoimmune OR 'auto immune' OR 'insulin dependent')):ab,ti,kw<br>3) 'incidence'/de<br>4) (incidence OR registry):ab,ti,kw<br>5) 'infant'/exp OR 'preschool child'/de OR 'child'/de OR 'adolescence'/exp OR 'young adult'/de<br>6) (neonat* OR 'new born*' OR newborn* OR infancy OR infant OR infants OR 'pre school' OR preschool OR preschooler* OR child* OR 'school age*' OR schoolage* OR preadolescen* OR puberty OR adolescen* OR 'early adulthood' OR 'young adult*'):ab,ti,kw<br>(1 OR 2) AND (3 OR 4) AND (5 OR 6)
(c) Global Health (EBSCO, C.A.B. International) = 636 results 04/12/2021<br>Settings: Advanced search<br>remove: Apply equivalent subjects<br>Published Dates = 1990 – 2021; Publication type = academic journal<br>DE "type 1 diabetes"<br>OR TITLE OR ABSTRACT:<br>(diabetes N1 ("type 1" OR "type I" OR childhood OR "juvenile onset" OR autoimmune OR "auto immune" OR "insulin dependent"))<br>AND<br>DE "incidence" OR DE "disease incidence"<br>OR TITLE OR ABSTRACT:<br>incidence OR registry<br>AND<br>DE "neonates" OR DE "infants" OR DE "preschool children" OR DE "children" OR DE "adolescents" OR DE "young adults"<br>OR TITLE OR ABSTRACT:<br>neonat* OR "new born*" OR newborn* OR infancy OR infant OR infants OR "pre school" OR preschool OR preschooler* OR child* OR "school age*" OR schoolage* OR preadolescen* OR puberty OR adolescen* OR "early adulthood"' OR "young adult*"

Table C: Data extraction template

Field Group	***Field***	***Description***	***Notes***
**Study ID**	**Source**	Short citation: Last name of first author and year of publication	If Study ID (Name + Year) is not unique, add "a", "b" etc
**Demographics**	**Country**	Name of country	
	**Study Location**	Location within the country: Nationwide, region, city, etc.	
	**Years**	Years of the study	If multiple years are reported separately, extract estimates for each time period
	**Age Range**	Age range of study participants (years)	If multiple age groups are reported separately, extract estimates for each age group
**Estimates**	**Population**	Population size at risk (denominator)	Sex-specific if available
	**Cases of Diabetes**	Number of incident diabetes cases reported	Sex-specific if available
	**Incidence Rate (per 100,000)**	Incidence rate and 95% Cis (Upper and lower bound values)	To be extracted if cases and population size are not available. Sex-specific if available
**Study Details**	**Data Source**	Type of data collection: Population-based registry, facility registry, cohort study, etc.	Can be listed in the first row only for each source
	**Notes**	Other relevant notes	
	**PMID**	PMID (if available)	
	**URL**	URL of article	
	**Citation**	Full citation	

Preferred Reporting Items for Systematic reviews and Meta-Analyses extension for Scoping Reviews (PRISMA-ScR) Checklist

SECTION	ITEM	PRISMA-ScR CHECKLIST ITEM	REPORTED ON PAGE #
**TITLE**			
Title	1	Identify the report as a scoping review.	1
**ABSTRACT**			
Structured summary	2	Provide a structured summary that includes (as applicable): background, objectives, eligibility criteria, sources of evidence, charting methods, results, and conclusions that relate to the review questions and objectives.	3
**INTRODUCTION**			
Rationale	3	Describe the rationale for the review in the context of what is already known. Explain why the review questions/objectives lend themselves to a scoping review approach.	5,6
Objectives	4	Provide an explicit statement of the questions and objectives being addressed with reference to their key elements (e.g., population or participants, concepts, and context) or other relevant key elements used to conceptualize the review questions and/or objectives.	7
**METHODS**			
Protocol and registration	5	Indicate whether a review protocol exists; state if and where it can be accessed (e.g., a Web address); and if available, provide registration information, including the registration number.	8
Eligibility criteria	6	Specify characteristics of the sources of evidence used as eligibility criteria (e.g., years considered, language, and publication status), and provide a rationale.	8,9
Information sources*	7	Describe all information sources in the search (e.g., databases with dates of coverage and contact with authors to identify additional sources), as well as the date the most recent search was executed.	8,9
Search	8	Present the full electronic search strategy for at least 1 database, including any limits used, such that it could be repeated.	8
Selection of sources of evidence†	9	State the process for selecting sources of evidence (i.e., screening and eligibility) included in the scoping review.	9
Data charting process‡	10	Describe the methods of charting data from the included sources of evidence (e.g., calibrated forms or forms that have been tested by the team before their use, and whether data charting was done independently or in duplicate) and any processes for obtaining and confirming data from investigators.	9,10
Data items	11	List and define all variables for which data were sought and any assumptions and simplifications made.	9,10
Critical appraisal of individual sources of evidence§	12	If done, provide a rationale for conducting a critical appraisal of included sources of evidence; describe the methods used and how this information was used in any data synthesis (if appropriate).	9

SECTION	ITEM	PRISMA-ScR CHECKLIST ITEM	REPORTED ON PAGE #
Synthesis of results	13	Describe the methods of handling and summarizing the data that were charted.	11
**RESULTS**			
Selection of sources of evidence	14	Give numbers of sources of evidence screened, assessed for eligibility, and included in the review, with reasons for exclusions at each stage, ideally using a flow diagram.	11
Characteristics of sources of evidence	15	For each source of evidence, present characteristics for which data were charted and provide the citations.	11
Critical appraisal within sources of evidence	16	If done, present data on critical appraisal of included sources of evidence (see item 12).	-
Results of individual sources of evidence	17	For each included source of evidence, present the relevant data that were charted that relate to the review questions and objectives.	12
Synthesis of results	18	Summarize and/or present the charting results as they relate to the review questions and objectives.	13
**DISCUSSION**			
Summary of evidence	19	Summarize the main results (including an overview of concepts, themes, and types of evidence available), link to the review questions and objectives, and consider the relevance to key groups.	14-17
Limitations	20	Discuss the limitations of the scoping review process.	18
Conclusions	21	Provide a general interpretation of the results with respect to the review questions and objectives, as well as potential implications and/or next steps.	18
**FUNDING**			
Funding	22	Describe sources of funding for the included sources of evidence, as well as sources of funding for the scoping review. Describe the role of the funders of the scoping review.	19

JBI = Joanna Briggs Institute; PRISMA-ScR = Preferred Reporting Items for Systematic reviews and Meta-Analyses extension for Scoping Reviews.
* Where *sources of evidence* (see second footnote) are compiled from, such as bibliographic databases, social media platforms, and Web sites.
† A more inclusive/heterogeneous term used to account for the different types of evidence or data sources (e.g., quantitative and/or qualitative research, expert opinion, and policy documents) that may be eligible in a scoping review as opposed to only studies. This is not to be confused with *information sources* (see first footnote).
‡ The frameworks by Arksey and O'Malley (6) and Levac and colleagues (7) and the JBI guidance (4, 5) refer to the process of data extraction in a scoping review as data charting.
§ The process of systematically examining research evidence to assess its validity, results, and relevance before using it to inform a decision. This term is used for items 12 and 19 instead of "risk of bias" (which is more applicable to systematic reviews of interventions) to include and acknowledge the various sources of evidence that may be used in a scoping review (e.g., quantitative and/or qualitative research, expert opinion, and policy document).

From: Tricco AC, Lillie E, Zarin W, O'Brien KK, Colquhoun H, Levac D, et al. PRISMA Extension for Scoping Reviews (PRISMAScR): Checklist and Explanation. Ann Intern Med. 2018;169:467–473. doi: 10.7326/M18-0850.
