# Supplementary material for: Variation in the incidence of type 1 diabetes mellitus in children and adolescents by world region and country income group: A scoping review
Source: PLOS Glob Public Health. 2022 Nov 9;2(11):e0001099. doi: 10.1371/journal.pgph.0001099 (PMC10021400; doi:10.1371/journal.pgph.0001099)
Supplement: Attachment — Submitted filename: Response to Reviewers_v1.docx [file pgph.0001099.s002.docx]

**We thank the reviewers for their thoughtful comments, and have responded to all points below (in bold), with revisions to the manuscript highlighted in blue.**

**We have also provided relevant references wherever necessary and corrected minor grammatical errors. We have included additional lines on the research implications especially in reference to sub- Saharan African countries to improve research, databases and data sharing principles.**

**REVISIONS REQUIRED:**

==============================

On page 16, paragraph 1, the authors start the sentence by “What explains the wider variability…”- while there is no harm starting the paragraph with a question, the sentence structure here is poorly done. Authors may need to restructure.

Also, while authors mention that access to healthcare in high income countries is key in timely diagnosis of T1D, and that strengthened healthcare system etc is important in ensuring access to data, authors need to expound further why lack of these two is an impediment in getting accurate or timely information on the T1D incidence in many LMICs.

I like how the author speculate that it may be possible that many children die earlier in SSA before diagnosis. Can authors build up on this and add relevant references.

Also, authors have associated lifestyle changes and obesity cases with T1D in developed countries. However, we know that under nutrition as well as eating unhealthy diets and physical inactivity are major issues contributing to NCDs in LMIC today. Can the author substantiate on this by comparing developed and developing countries?

Authors have mentioned few policy implications for this work. I feel this study is very important and authors should add a paragraph or so on the research implications especially in African countries. Also, we know that LMIcs are lagging behind in terms of research, databases, and data sharing principles. What need to be done?
==============================

**RESPONSE TO REQUIRED REVISIONS:**

**Thank you for your valuable suggestions and comments.**

**As part of our revisions we have modified the sentence regarding the wider variability (Line 282-283 , Page 16).**

**Page 16, Line 282-283:**

Globally, we find wide variability in incidence of T1D and higher estimates than recent estimates from IDF Diabetes Atlas.^1^

**Addressing to the comment on timely diagnosis of T1D and lack of strengthened health care system, we have included an explanation and relevant references (Lines 251-266) to emphasize the need of getting accurate and timely information on T1D incidence in many LMICs.**

**We have also made minor change by replacing ‘type 1 diabetes’ with an acronym ‘T1D’ to ensure consistency and clarity throughout the paper.**

**Page 15, Lines 251 - 266:**

Studies have also found that for some children an accurate T1DM diagnosis is initially missed by clinicians even in some high-income settings.^16,30–33^ For example, a patient survey in the United States found that 25% all patients reported being initially misdiagnosed with another condition and misdiagnosis was associated with 18% increased risk of DKA than those correctly diagnosed.^34^ The potential barriers in receiving timely diagnosis as explored in the survey included lack of primary care provider, lack of time, lack of insurance, having a high deductible or copay, difficulty getting an appointment with a physician, lack of transportation etc. In many LMICs, levels of misdiagnosis are likely higher, with studies finding that in sub-Saharan Africa most healthcare workers are not able to timely recognize symptoms and signs of DKA which lead to high mortality.^35,36^ Indeed, many children presenting with diabetic coma in Africa are likely to be treated for more common diseases such as cerebral malaria or meningitis before the correct diagnosis is suspected.^37^ It has therefore long been suspected that many children in such settings die before being correctly diagnosed with T1D. This helps explaining the paucity of data from many LMICs and emphasizes on the importance of accurate and timely diagnosis in these settings.

**We have also included the following additional relevant references (in support of lines 251-266) in the manuscript to provide evidence that it may be possible that many children die earlier in SSA before diagnosis.**

1. **Ward ZJ, Yeh JM, Bhakta N, Frazier AL, Atun R. Estimating the total incidence of global childhood cancer: a simulation-based analysis. Lancet Oncol. 2019 Apr;20(4):483-493.**
2. **Atun R, Bhakta N, Denburg A, et al.. Sustainable care for children with cancer: a Lancet Oncology Commission. Lancet Oncol. 2020 Apr;21(4):e185-e224.**
3. **Ward Z, Yeh J, Reddy CL et al. Estimating the total incidence of type 1 diabetes in children and adolescents 0-19 from 1990-2050: a global simulation-based analysis. Lancet Diabetes and Endocrinology (In press)**
4. **Muñoz C, Floreen A, Garey C, Karlya T, Jelley D, Alonso GT, McAuliffe-Fogarty A. Misdiagnosis and Diabetic Ketoacidosis at Diagnosis of Type 1 Diabetes: Patient and Caregiver Perspectives. *Clin Diabetes* 2019; 37(3): 276-281.**
5. **Monabenka HG, Mbika-Cardorelle A, Moyen G. Ketoacidosis in children and teenagers in Congo. *Sante* 2003; 13: 139-141.**
6. **Majaliwa ES, Mohn A, Chiavaroli V, Ramaiya K, Swai AB, Chiarelli F. Management of diabetic ketoacidosis in children and adolescents in sub-Saharan Africa: a review. *East Afr Med J* 2010; 87(4): 167-73.**
7. **Rwitza HT, Swai ABM, McLary DG. Failure to diagnose diabetic ketoacidosis in Tanzania. *Diabetic Medicine* 1986; 35: 645–648.**

**Thank you for your comment referring to the concerns of associated lifestyle changes and obesity cases with T1D in developed countries versus unhealthy diets and lack of physical activities for developing countries.**

**To further substantiate we have included lines 274-280 to explain the association of autoimmunity with elevated BMI and sedentary lifestyle which holds true in both developed and developing countries.**

**Page 16, Lines 274-280:**

A positive relationship exists between a country’s level of economic development as measured by GDP and obesity.^40^ An elevated BMI and a sedentary lifestyle may exacerbate insulin resistance, which could lead to $\beta$-cells fatigue and triggering of an autoimmune response with resultant $\beta$-cell apoptosis.^41,42^ There exist a complex relationship between environmental factors and genetic risk for T1D or type 2 diabetes which likely plays a role in autoimmunity pathogenesis and presentation of clinical disease.

**To further explain this association is beyond the scope of our manuscript. We have provided the following explanation below as our response to avoid exceeding the word limit of the manuscript and in the interest of time of our valuable readers.**

**Recent research has reported that obesity has long been recognized as a major risk factor for the development of type 2 diabetes and a catalyst for complications, much less is known about the role of obesity in the initiation and pathogenesis of type 1 diabetes. The following study explains the rising association between obesity and type 1 diabetes and “accelerator hypothesis” which is beyond the scope of our current manuscript hence hasn’t been included much in detail. The “accelerator hypothesis” proposes that obesity-induced insulin resistance places increased burden on islets in genetically at-risk individuals, inducing autoimmune destruction and/or accelerating its course for type 1 diabetes. This is beyond the scope of our research and hasn’t been explained in much detail in the manuscript.**

**Karen D Corbin, Kimberly A Driscoll, Richard E Pratley, Steven R Smith, David M Maahs, Elizabeth J Mayer-Davis, Advancing Care for Type 1 Diabetes and Obesity Network (ACT1ON), Obesity in Type 1 Diabetes: Pathophysiology, Clinical Impact, and Mechanisms, Endocrine Reviews, Volume 39, Issue 5, October 2018, Pages 629–663,** [**https://doi.org/10.1210/er.2017-00191**](https://doi.org/10.1210/er.2017-00191)

**We appreciate your comment regarding future policy implications and what needs to be done. We have included additional lines (Page 17, Lines 308-310 and Page 18, lines 328-332) to suggest some future research implications especially in the African context.**

**Policy-makers should allocate appropriate levels of resources and plan for adequate diagnostic and medical capacity as part of efforts to improve detection, given the large proportion of cases that are not currently diagnosed and the projected rise in the incidence of T1D around the world, with especially high numbers of new cases projected to occur in Africa, where investments are needed to strengthen health systems and achieve universal health coverage.**

**Page 17, Lines 308-310**

Simulation-based analysis estimates that by 2050, the total number of new cases of T1D are projected to increase especially in Africa which will account for 51% of global new cases of T1D per year and highest DKA admissions at diagnosis.^54^

**Page 18, Lines 328-332**

One such example could be diagonally integrating diabetes care into the broader health system in order to improve the quality of care for T1D and also other chronic illnesses.^56,57^ This can include policy prioritization, innovative financing, task-shifting, secure and sustainable access to essential medicines and diagnostic tools, and comprehensive service delivery.

**The following relevant references have been included in the manuscript to support the above lines:**

**54. Ward ZJ. Estimating the total incidence of type 1 diabetes in children and adolescents 0-19 from 1990-2050: a global simulation-based analysis. *Lancet Diabetes and Endocrinology (In press)*.**

**55. Atun R, Davies JI, Gale EAM, et al. Diabetes in sub-Saharan Africa: from clinical care to health policy. *Lancet Diabetes Endocrinol*. 2017;5(8):622-667. doi:10.1016/S2213-8587(17)30181-X**

**56. Knaul FM, Bhadelia A, Atun R, Frenk J. Achieving Effective Universal Health Coverage And Diagonal Approaches To Care For Chronic Illnesses. *Health Affairs*. 2015;34(9):1514-1522. doi:10.1377/hlthaff.2015.0514**

**57. Atun R, Jaffar S, Nishtar S, et al. Improving responsiveness of health systems to non-communicable diseases. *The Lancet*. 2013;381(9867):690-697. doi:10.1016/S0140-6736(13)60063-X**

**In reference to the figure legend for Figure 1 (Supplementary Figure):**

Figure 1 (Supplementary Figure): please (a) provide a direct link to the base layer of the map used and ensure this is also included in the figure legend; (b) provide a link to the terms of use / license information for the base layer. We cannot publish proprietary or copyrighted maps (e.g. Google Maps, Mapquest) and the terms of use for your map base layer must be compatible with our CC-BY 4.0 license.

If your map was obtained from a copyrighted source please amend the figure so that the base map used is from an openly available source. Alternatively, please provide explicit written permission from the copyright holder granting you the right to publish the material under our CC-BY 4.0 license.Please note that the following CC BY licenses are compatible with PLOS license: CC BY 4.0, CC BY 2.0 and CC BY 3.0, meanwhile such licenses as CC BY-ND 3.0 and others are not compatible due to additional restrictions. If you are unsure whether you can use a map or not, please do reach out and we will be able to help you. The following websites are good examples of where you can source open access or public domain maps:

* U.S. Geological Survey (USGS) - All maps are in the public domain. ([http://www.usgs.gov](http://www.usgs.gov/))

* PlaniGlobe - All maps are published under a Creative Commons license so please cite “PlaniGlobe, [http://www.planiglobe.com](http://www.planiglobe.com/), CC BY 2.0” in the image credit after the caption. (<http://www.planiglobe.com/?lang=enl>)

* Natural Earth - All maps are public domain. (<http://www.naturalearthdata.com/about/terms-of-use/>)

**We have provided a direct link to the base layer of the map used this is also included in the figure legend.**

**The map was made in R statistical computing package with the maps package which is GPL-2 :** [**https://cran.r-project.org/web/packages/maps/index.html**](https://cran.r-project.org/web/packages/maps/index.html) **and uses the Natural Earth map data.**

==============================

**RESPONSE TO REVIEWERS' COMMENTS:**

Reviewer #1: Dear Authors,

This study is a scoping literature review on the incidence of type 1 diabetes in children, adolescents, and young adults (0-25 years) worldwide and explains the wide heterogeneity in incidence rates that differ by age, sex, world region, and country income classification. I took keen interest to read through this important study , which I find to be timely especially given that incidences of T1D globally and in SSA are not well documented. The authors did a great job in categorizing their analysis and results in terms of region, age etc and also used the IDF age classification to conduct their analysis. Findings from this study are important not only to readers of Plos GPH, but also scientists, policy makers and other stakeholders interested in the field of diabetes broadly and T1D specifically.

The introduction section is well summarized and the gap is well stated. However, authors should ensure that they include relevant references where necessary. For example, paragraph 1, after LMIC, authors should add a reference.
The methodology section is well explained. The authors have followed key steps of scoping review and processes of the scoping review are well presented. This study can easily be replicated by other researchers.
Results and discussion: these sections are presented in a logical fashion. The presentation is also easier to understand. Statistics and presentations are well done .
Overall, the writing style is good and in standard English. The language is consistent, short and meaningful paragraphs that makes it easy for the reader to follow the narrative.

I have few things that the may consider correcting to strengthen their manuscript:
On page 16, paragraph 1, the authors start the sentence by “What explains the wider variability…”- while there is no harm starting the paragraph with a question, the sentence structure here is poorly done. Authors may need to restructure.
Also, while authors mention that access to healthcare in high income countries is key in timely diagnosis of T1D, and that strengthened healthcare system etc is important in ensuring access to data, authors need to expound further why lack of these two is an impediment in getting accurate or timely information on the T1D incidence in many LMICs. I like how the author speculate that it may be possible that many children die earlier in SSA before diagnosis. Can authors build up on this and add relevant references.
Also, authors have associated lifestyle changes and obesity cases with T1D in developed countries. However, we know that under nutrition as well as eating unhealthy diets and physical inactivity are major issues contributing to NCDs in LMICs today. Can the author substantiate on this by comparing developed and developing countries?
Authors have mentioned few policy implications for this work. I feel this study is very important and authors should add a paragraph or so on the research implications especially in African countries. Also, we know that LMICs are lagging behind in terms of research, databases, and data sharing principles. What need to be done?

**We highly appreciate your support and comments on the scoping review. We have incorporated all your suggestion into our revisions. We have provided relevant references wherever necessary and also included the reference to the highlighted comment on LMIC in paragraph 1. The other comments have been explained as described above (included in the section for revisions required).**

Reviewer #2:

I thank the authors for sharing their work for publication.
In the present study, the authors have attempted to assess the Type 1 Diabetes Mellitus Incidence in Children and Adolescents Variation by World Region and Country Income Group. The review concludes T1D incidence rates vary significantly by geographic region, country economic level, and age.
Overall the study offers an important piece of information.
1. The title properly reflects the subject of the paper.
2. The abstract is short, accurate and clear. It provides an accessible summary of the paper.
3. The keywords accurately reflect the content.
4. Introduction is well written and Summarizes recent research related to the topic
5. The methods are replicable and follows the best practice standards
6. The result seems plausible and the study provides sufficient data, clear data tables and non-contradictory data that agree with the conclusions.
7. The referencing is accurate, adequate and balanced.

**Thank you for your comments and appreciate your time in reviewing our manuscript.**

**JOURNAL REQUIREMENTS:**

1. Please amend your online Financial Disclosure statement. If you did not receive any funding for this study, please simply state: “The authors received no specific funding for this work.”

Included in the online Financial disclosure statement.

2. Please update your online Competing Interests statement. If you have no competing interests to declare, please state: “The authors have declared that no competing interests exist.”

Included in the online competing interest statement.

3. Please provide a complete Data Availability Statement in the submission form, ensuring you include all necessary access information or a reason for why you are unable to make your data freely accessible. If your research concerns only data provided within your submission, please write “All data are in the manuscript and/or supporting information files.” as your Data Availability Statement.

Included the updated sentences in the online data availability statement.

4. We have noticed that you have uploaded Supporting Information files, but you have not included a list of legends. Please add a full list of legends for your Supporting Information files after the references list.

List of legends for supporting information files has been included after the references list

5. All figures and supporting information files will be published under the Creative Commons Attribution License ([creativecommons.org/licenses/by/4.0/](http://creativecommons.org/licenses/by/4.0/)). Authors retain ownership of the copyright for their article and are responsible for third-party content used in the article.

Figure 1 (Supplementary Figure): please (a) provide a direct link to the base layer of the map used and ensure this is also included in the figure legend; (b) provide a link to the terms of use / license information for the base layer. We cannot publish proprietary or copyrighted maps (e.g. Google Maps, Mapquest) and the terms of use for your map base layer must be compatible with our CC-BY 4.0 license.

If your map was obtained from a copyrighted source please amend the figure so that the base map used is from an openly available source. Alternatively, please provide explicit written permission from the copyright holder granting you the right to publish the material under our CC-BY 4.0 license.

Please note that the following CC BY licenses are compatible with PLOS license: CC BY 4.0, CC BY 2.0 and CC BY 3.0, meanwhile such licenses as CC BY-ND 3.0 and others are not compatible due to additional restrictions.

If you are unsure whether you can use a map or not, please do reach out and we will be able to help you. The following websites are good examples of where you can source open access or public domain maps: 
* U.S. Geological Survey (USGS) - All maps are in the public domain. (<http://www.usgs.gov>) 
* PlaniGlobe - All maps are published under a Creative Commons license so please cite “PlaniGlobe, <http://www.planiglobe.com>, CC BY 2.0” in the image credit after the caption. (<http://www.planiglobe.com/?lang=enl>) 
* Natural Earth - All maps are public domain. (<http://www.naturalearthdata.com/about/terms-of-use/>)

Please upload any written confirmation as an 'Other' file type. It must clarify that the copyright holder understands and agrees to the terms of the CC BY 4.0 license; general permission forms that do not specify permission to publish under the CC BY 4.0 will not be accepted. Note that uploading an email confirmation is acceptable.

6. Please review your reference list to ensure that it is complete and correct. If you have cited papers that have been retracted, please include the rationale for doing so in the manuscript text, or remove these references and replace them with relevant current references. Any changes to the reference list should be mentioned in the rebuttal letter that accompanies your revised manuscript. If you need to cite a retracted article, indicate the article’s retracted status in the References list and also include a citation and full reference for the retraction notice.

Additional Editor Comments (if provided):


[Note: HTML markup is below. Please do not edit.]

Reviewers' comments:

Reviewer's Responses to Questions

**Comments to the Author**

1. Does this manuscript meet PLOS Global Public Health’s [publication criteria](https://journals.plos.org/globalpublichealth/s/criteria-for-publication)? Is the manuscript technically sound, and do the data support the conclusions? The manuscript must describe methodologically and ethically rigorous research with conclusions that are appropriately drawn based on the data presented.

Reviewer #1: Yes

Reviewer #2: Yes

2. Has the statistical analysis been performed appropriately and rigorously?

Reviewer #1: Yes

Reviewer #2: Yes

3. Have the authors made all data underlying the findings in their manuscript fully available (please refer to the Data Availability Statement at the start of the manuscript PDF file)?

The [PLOS Data policy](https://journals.plos.org/globalpublichealth/s/data-availability) requires authors to make all data underlying the findings described in their manuscript fully available without restriction, with rare exception. The data should be provided as part of the manuscript or its supporting information, or deposited to a public repository. For example, in addition to summary statistics, the data points behind means, medians and variance measures should be available. If there are restrictions on publicly sharing data—e.g. participant privacy or use of data from a third party—those must be specified.

Reviewer #1: No

Reviewer #2: Yes

4. Is the manuscript presented in an intelligible fashion and written in standard English?

PLOS Global Public Health does not copyedit accepted manuscripts, so the language in submitted articles must be clear, correct, and unambiguous. Any typographical or grammatical errors should be corrected at revision, so please note any specific errors here.

Reviewer #1: Yes

Reviewer #2: Yes

5. Review Comments to the Author

Please use the space provided to explain your answers to the questions above. You may also include additional comments for the author, including concerns about dual publication, research ethics, or publication ethics. (Please upload your review as an attachment if it exceeds 20,000 characters)

Reviewer #1: Dear Authors,

This study is a scoping literature review on the incidence of type 1 diabetes in children, adolescents, and young adults (0-25 years) worldwide and explains the wide heterogeneity in incidence rates that differ by age, sex, world region, and country income classification. I took keen interest to read through this important study , which I find to be timely especially given that incidences of T1D globally and in SSA are not well documented. The authors did a great job in categorizing their analysis and results in terms of region, age etc and also used the IDF age classification to conduct their analysis. Findings from this study are important not only to readers of Plos GPH, but also scientists, policy makers and other stakeholders interested in the field of diabetes broadly and T1D specifically.

The introduction section is well summarized and the gap is well stated. However, authors should ensure that they include relevant references where necessary. For example, paragraph 1, after LMIC, authors should add a reference.
The methodology section is well explained. The authors have followed key steps of scoping review and processes of the scoping review are well presented. This study can easily be replicated by other researchers.
Results and discussion: these sections are presented in a logical fashion. The presentation is also easier to understand. Statistics and presentations are well done .
Overall, the writing style is good and in standard English. The language is consistent, short and meaningful paragraphs that makes it easy for the reader to follow the narrative.
I have few things that the may consider correcting to strengthen their manuscript:
On page 16, paragraph 1, the authors start the sentence by “What explains the wider variability…”- while there is no harm starting the paragraph with a question, the sentence structure here is poorly done. Authors may need to restructure.
Also, while authors mention that access to healthcare in high income countries is key in timely diagnosis of T1D, and that strengthened healthcare system etc is important in ensuring access to data, authors need to expound further why lack of these two is an impediment in getting accurate or timely information on the T1D incidence in many LMICs. I like how the author speculate that it may be possible that many children die earlier in SSA before diagnosis. Can authors build up on this and add relevant references.
Also, authors have associated lifestyle changes and obesity cases with T1D in developed countries. However, we know that under nutrition as well as eating unhealthy diets and physical inactivity are major issues contributing to NCDs in LMICs today. Can the author substantiate on this by comparing developed and developing countries?
Authors have mentioned few policy implications for this work. I feel this study is very important and authors should add a paragraph or so on the research implications especially in African countries. Also, we know that LMICs are lagging behind in terms of research, databases, and data sharing principles. What need to be done?

Reviewer #2: I thank the authors for sharing their work for publication.
In the present study, the authors have attempted to assess the Type 1 Diabetes Mellitus Incidence in Children and Adolescents Variation by World Region and Country Income Group. The review concludes T1D incidence rates vary significantly by geographic region, country economic level, and age.
Overall the study offers an important piece of information.
1. The title properly reflects the subject of the paper.
2. The abstract is short, accurate and clear. It provides an accessible summary of the paper.
3. The keywords accurately reflect the content.
4. Introduction is well written and Summarizes recent research related to the topic
5. The methods are replicable and follows the best practice standards
6. The result seems plausible and the study provides sufficient data, clear data tables and non-contradictory data that agree with the conclusions.
7. The referencing is accurate, adequate and balanced.

6. PLOS authors have the option to publish the peer review history of their article ([what does this mean?](https://journals.plos.org/globalpublichealth/s/editorial-and-peer-review-process#loc-peer-review-history)). If published, this will include your full peer review and any attached files.


**Do you want your identity to be public for this peer review?** If you choose “no”, your identity will remain anonymous but your review may still be made public.


For information about this choice, including consent withdrawal, please see our [Privacy Policy](https://www.plos.org/privacy-policy).

Reviewer #1: No

Reviewer #2: No
